# Cystic Echinococcosis in Northern New Hampshire, USA

**DOI:** 10.3201/eid2905.221828

**Published:** 2023-05

**Authors:** Ahmad AlSalman, Abigail Mathewson, Isabella W. Martin, Rattanaporn Mahatanan, Elizabeth A. Talbot

**Affiliations:** Dartmouth Health, Lebanon, New Hampshire, USA (A. AlSalman, I.W. Martin, R. Mahatanan, E.A. Talbot);; Dartmouth College, Hanover, New Hampshire, USA (A. AlSalman, I.W. Martin, R. Mahatanan, E.A. Talbot);; New Hampshire Department of Health and Human Services, Concord, New Hampshire, USA (A. Mathewson, E.A. Talbot)

**Keywords:** echinococcosis, parasites, *Echinococcus granulosus*, pulmonary echinococcosis, cystic echinococcosis, zoonoses, New Hampshire, United States

## Abstract

In April 2022 and December 2022, the New Hampshire Department of Health and Human Services confirmed 2 cases of locally acquired human pulmonary cystic echinococcosis caused by *Echinococcus granulosus* tapeworms. Both patients reported dressing locally hunted moose and exposure to dogs.

*Echinococcus granulosus* is a zoonotic cestode that lives in the intestine of its definitive host, canids (e.g., dogs, coyotes, foxes, wolves). Embryonated eggs released in canid feces are immediately infectious when consumed by an intermediate host, usually an ungulate (e.g., moose, deer, sheep) or sometimes a human (aberrant intermediate host). An ingested egg hatches in the small intestine, becoming an oncosphere that travels to organs such as the lung, liver, spleen, bone, or brain where it forms a thick-walled cyst. Canids become infected when they ingest flesh and viscera of an infected intermediate host (*1*). We report 2 human cases of *E. granulosus* tapeworm infection in northern New Hampshire, USA, during 2022.

In April 2022, an otherwise healthy middle-aged man (patient 1) from rural northern New Hampshire sought care from his primary-care physician for a routine physical examination. On chest auscultation, the physician heard localized wheezes, prompting chest radiograph and subsequent computed tomography scan; the images showed a noncalcified 4-cm mass in the lower lobe of the right lung. After initial endobronchial biopsy of the mass was nondiagnostic, the patient underwent thoracoscopic lung wedge resection. A gray cystic lesion ruptured during the procedure, releasing clear fluid. The ruptured cyst was excised, and histologic examination showed the presence of a laminated outer cyst wall and numerous daughter cysts, each containing multiple protoscolices with internal hooklets and calcareous corpuscles (Figure), features diagnostic of *E. granulosus* tapeworm infection. The patient was prescribed a treatment course of albendazole and planned close follow-up, given the high risk for recurrence.

**Figure:**
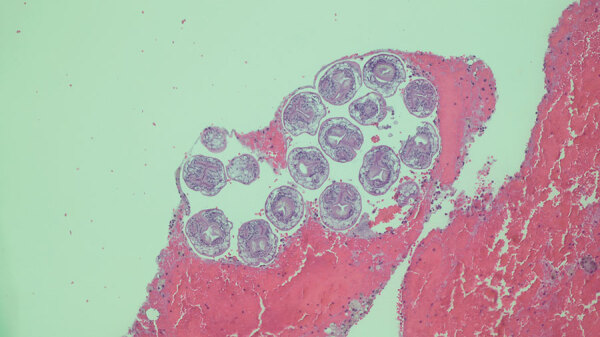
Lung biopsy specimen from a patient with *Echinococcus granulosus* tapeworm infection shows a daughter cyst containing multiple protoscolices with internal hooklets and calcareous corpuscles. Hematoxylin and eosin stain; original magnification ×100.

In December 2022, patient 2, an otherwise healthy middle-aged woman, sought medical attention for anaphylaxis that was determined to be caused by a ruptured cyst. Radiography showed 2 lung lesions and 1 small (<5 cm) liver lesion. Results of serum *Echinococcus* antibody testing was positive. A course of albendazole was prescribed (400 mg 2×/d) and, 4 weeks into therapy, the patient had the first larger pulmonary lesion successfully excised with plans for resection of the second excision.

Both patients reported similar epidemiologic risk factors of dressing locally hunted moose and being exposed to dogs in northern New Hampshire. Neither patient reported relevant travel history outside of New Hampshire. Patient 1 reported observing that the proportion of moose carcasses with extensive cystic lung lesions has increased in recent years. The stool of his 4-year-old domestic dog tested negative for *E. granulosus* through a commercial veterinary laboratory. We postulate that both patients acquired *E. granulosus* infection either by consuming produce contaminated with infected canid feces or through consumption of eggs shed in their dogs’ stool. The patients gave verbal permission to publish this report.

In the United States, locally acquired human *E. granulosus* infections remain rare. However, cases in Arctic and sub-Arctic areas of Canada and Alaska are well described, mainly among Native American populations (*2*–*4*). In the contiguous United States, a few human cases are reported annually in Arizona, New Mexico, California, and Utah (*4*). *E. granulosus* infections have been described in wildlife of northern New England (*5*,*6*); in 2012, surveillance of the hunter-harvested moose population (*Alces alces*) in Maine documented the presence of the G8 genotype *E. granulosus* (Canadensis) in 21/54 (39%) of lung sets examined. Domestic dogs and eastern coyotes (*Canis latrans*) were suggested as definitive hosts, given the absence of wolves in the area (*5*). Other areas in the contiguous United States where sylvatic cycles of transmission have been confirmed include Idaho, Minnesota, California, and Montana; stable cycles were described mainly in the Arctic and sub-Arctic areas of Canada and Alaska (*2*). Information about *E. granulosus* infections in local wild canids in northern New England is lacking.

New Hampshire Department of Health and Human Services has notified healthcare providers caring for patients involved in moose hunting about preventive measures. Such measures include wearing waterproof gloves when dressing moose carcasses, preventing domestic dogs and wild canids from consuming raw viscera, disposing of all viscera in accordance with recommendations from New Hampshire Fish and Game Department, thoroughly cooking all game meat that is fed to dogs, and administering to dogs a deworming agent effective against tapeworms at least twice per year.
